# Role of μ-opioid receptor in parafascicular nucleus of thalamus on morphine-induced antinociception in a rat model of acute trigeminal pain

**Published:** 2017-03-15

**Authors:** Esmaeal Tamaddonfard, Amir Erfanparast

**Affiliations:** *Department of Basic Sciences, Faculty of Veterinary Medicine, Urmia University, Urmia, Iran.*

**Keywords:** Acute trigeminal pain, Morphine, Parafascicular nucleus, Rat, μ-opioid receptor

## Abstract

The parafascicular nucleus (PFN) of thalamus, as a supraspinal structure, has an important role in processing of nociceptive information. In addition, μ-opioid receptor contributes to supraspinal modulation of nociception. In the present study, the effects of microinjection of naloxone (a non-specific opioid-receptor antagonist) and naloxonazine (a specific μ-opioid receptor antagonist) were investigated on morphine-induced antinociception in a rat model of acute trigeminal pain. Right and left sides of PFN of thalamus were implanted with two guide cannulas. Acute trigeminal pain was induced by local corneal surface application of hypertonic saline and the number of eye wipes as a pain index was recorded for 30 sec. Microinjection of morphine at doses of 1, 2 and 4 μg per site significantly (*p* < 0.05) decreased the number of eye wipes. Alone microinjection of naloxone (4 μg per site) and naloxonazine (1 and 2 μg per site) significantly (*p* < 0.05) increased corneal pain severity. Prior microinjection of naloxone (2 and 4 μg per site) and naloxonazine (1 and 2 μg per site) significantly (*p* < 0.05) prevented the antinociceptive effect induced by morphine (4 μg per site). All the above-mentioned chemicals did not alter locomotor behavior in an open-field test. The results of the present study showed an antinociceptive effect of morphine at the PFN level of thalamus. Mu-opioid receptor of the PFN of thalamus may be involved in morphine-induced antinociception.

## Introduction

Opioid system, through activation of specific μ-, δ- and κ-opioid receptors regulates many aspects of physiology and neurobiology such as memory, eating, seizures, thermoregulation and pain modulation.^1^ Morphine, as an opioid system agonist, affects peripheral, spinal and supra-spinal mechanisms of pain to produce antinociception and through a naloxone-sensitive mechanism inhibits the activity of cutaneous nociceptors under condition of inflammation.^2^ Intrathecal injection of morphine produces antinociceptive effects in the formalin test of rats.^3^ In addition, microinjection of morphine into the periaqueductal gray increases hot-plate latency in rats.^4^

Parafascisular nucleus (PFN), a posterior component of the intralaminar nuclei of the thalamus, plays an important role in the central processing and modulation of pain. Electrolytic lesions or local blocks of PFN by lidocaine produce transient but significant attenuation of the neuropathic manifestation in spared nerve injury model of mononeuropathy.^5^ In this context, the antinociceptive effects induced by intra-PFN microinjection of acetyl-choline and physostigmine have been inhibited by prior microinjection of atropine into the same sites in a rat model of acute corneal pain.^6^

Most studies have explored the peripheral and spinal cord mechanisms of morphine analgesia, whereas supra-spinal mechanisms are less investigated. Since opioid receptors are distributed in various nuclei of thalamus,^7^ and the thalamus has an important role in supraspinal modulation of pain,^8^^-^^10^ this study was aimed to investigate the role of these receptors in morphine-induced anti-nociception at the PFN level of thalamus using a rat model of acute trigeminal pain. For this purpose, microinjections of naloxone (a non-specific opioid-receptor antagonist) and naloxonazine (a specific μ-opioid receptor antagonist), alone and before morphine microinjection into PFN of thalamus were performed. Hypertonic saline-induced corneal pain, an acute trigeminal pain test, was introduced by Farazifard *et al*.^11^ It has been used for the study of the involvement of supraspinal mechanisms in acute trigeminal nociceptive modulation.^12^^,^^13^ For example, it has been reported that prior intracerebroventricular injection of ranitidine prevents histamine-induced antinociception in a rat model of acute corneal pain.^12^ Moreover, the involve-ment of muscarinic acetylcholine receptor of PFN has been reported in modulation of acute trigeminal pain in rats.^6^

## Materials and Methods

**Animals.** Healthy adult male Wistar rats (280 to 320 g) were used in this study. The animals were provided from animal house of Laboratory of Physiology of Faculty of Veterinary Medicine of Urmia University, Urmia, Iran. The rats were maintained in groups of six per cage in a light- dark cycle (light on at 07:00 AM) at a controlled ambient temperature (22 ± 0.5 ˚C) with *ad libitum* food and water access. All experiments were performed between 12:00 PM to 17: 00 PM. All research and animal care procedures were approved by the Veterinary Ethics Committee of Faculty of Veterinary Medicine of Urmia University. 

**Chemicals. **The chemicals used in the present study included morphine sulfate (Temad, Tehran, Iran), naloxone dihydrochloride and naloxonazine dihydro-chloride hydrate (Sigma-Aldrich Chemical Co., St. Louis, USA). All chemicals were dissolved in sterile normal saline. 

**Surgical procedure.** To deliver the compounds to be tested, each rat was anesthetized with intraperitoneal injection of a mixture of 80 mg kg^-1 ^ketamine (Alfasan, Woerden, Holland) and 8 mg kg^-1 ^xylazine (Alfasan) and then placed in a stereotaxic apparatus (Stoelting, Wood Lane, USA). Two 24-gauge, 15-mm length guide cannulas were bilaterally implanted 1 mm over the right and left sides of PFN at the following coordinates: 4.2 mm posterior to the bregma, 1.2 mm left and right sides of the midline and 6 mm below the top of the skull according to Paxinos and Watson and our previous study.^6^^,^^14^ The cannulas were then fixed to the skull using three screws and dental acrylic. A 29-gauge, 15 mm stylet was inserted into each cannula to keep them patent prior to microinjection. At least 10 days were allowed for recovery from the surgery.

**Intra-PFN microinjection **Bilateral intra-PFN micro-injections of normal saline (control), morphine at doses of 0.25, 0.5, 1, 2 and 4 μg per site, naloxone at doses of 1, 2 and 4 μg per site and naloxonazine at doses of 0.5, 1 and 2 μg per site were performed. In pretreatment schedule, prior microinjections of naloxone (2 and 4 μg per site) and naloxonazine (1 and 2 μg per site) before 4 μg per site microinjection of morphine were also done. All the above-mentioned chemicals were bilaterally administered using a 30-gauge, 16 mm needle attached to a 1 µL Hamilton syringe. A constant volume of 0.25 µL of the drug solution was microinjected into each PFN over a period of 60 sec. The injection needle was left in place for a further 60 s after completion of injection to facilitate diffusion of the drug. Naloxone and naloxonazine were microinjected six min and morphine was microinjected three min before induction of corneal pain. The drug doses used here were designed according to previous studies.^15^^-^^18^

**Acute trigeminal nociception. **This was induced by local corneal surface application of hypertonic saline. Briefly, rats were placed on wooden tables. After a 15 min adaptation period, one drop (40 µL) of a 5 M NaCl solution was locally applied on the corneal surface using a fine dropper. The number of eye wipes performed with the ipsilateral forepaw was counted for a period of 30 sec. Thereafter, the eye was washed by local application of distilled water on the corneal surface. All the observers were blinded to the protocol of the study. 

**Locomotor activity. **Five days after the end of pain study, locomotor activity was assessed in an open-field test as described previously.^18^ The apparatus consisted of a wooden box measuring 120 × 120 × 50 cm. The floor of the arena was divided into 16 equal squares. To monitor the activity, animals were removed from the home cage and placed directly into one corner of the open field apparatus. The number of squares crossed with all paws (line-crossings) and the number of rearing were counted in a 5-min session. 

**Cannula verification. **At the end of each experiment, 0.25 µL of methylene blue was injected into the each side of PFN. Animals were deeply anesthetized with the high dose ether and perfused intracardially with physiological saline followed by 10% formalin solution. The brains were removed and placed in the formalin. After 24 hr, the brains were sectioned coronally (100 and 200 µm) and viewed under a loupe to localize the injection site according to the atlas of Paxinos and Watson.^14^ The results obtained from five rats with guide cannulas outside the PFN were eliminated from the data analysis. 

**Statistical analysis. **Statistical comparisons were performed using GraphPad Prism (version 5; GraphPad software Inc., San Diego, USA). Data were analyzed using one-way ANOVA followed by Tukey’s test. Data are expressed as the mean ± SEM. Statistical significance was set at *p *< 0.05.

## Results

**Fig. 1:**
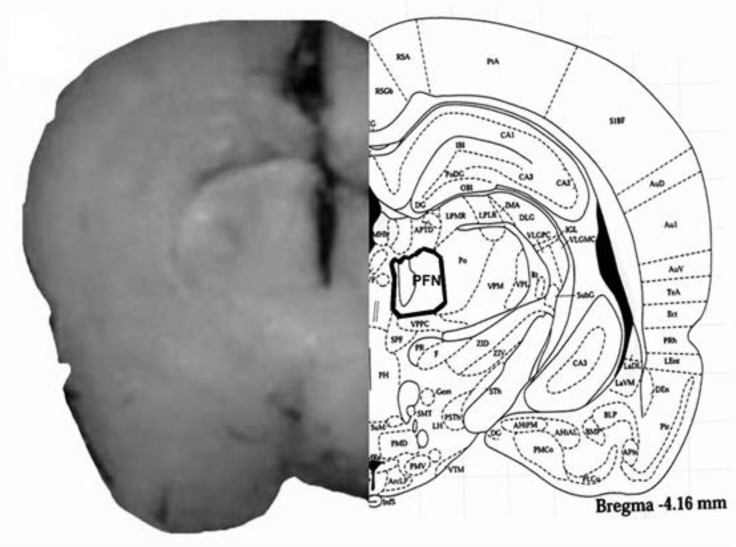
Schematic illustration of coronal section of the rat brain showing the approximate location of PFN microinjection sites in the experiments. Location of the injection cannulas tip in PFN (left side) of all rats was included in the data analysis. Atlas plate (right side) is adopted with permission from Paxinos and Watson

The placements of the tip of the cannulas in the PFN of rats are shown in Figure 1. The locations of the cannulas tip placements in the PFN were confirmed in the PFN sections (Fig. 1, Left side). The rat brain section (Fig. 1, Right side) was adopted from the atlas of Paxinos and Watson.^14^

**Fig. 2:**
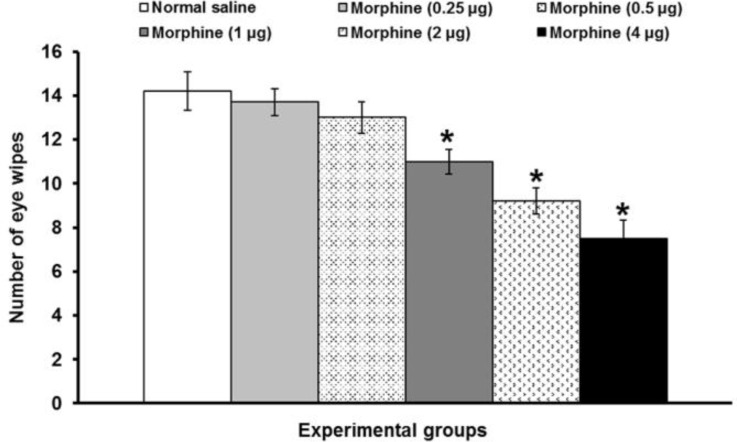
The effects of intra-PFN microinjection of morphine on corneal pain induced by topical corneal surface application of hypertonic saline. Data are the means ± SEM obtained from six rats. * *p *< 0.05 compared to normal saline microinjected group

The number of eye wipes after bilateral intra-PFN administration of normal saline was 14.2 ± 0.87. Intra-PFN microinjection of morphine at doses of 0.25 and 0.5 μg per site did not change the number of eye wipes induced by corneal surface application of hypertonic saline. Morphine at doses of 1, 2 and 4 μg per site significantly (*p* < 0.05) decreased the number of eye wipes (Fig. 2). 

**Fig. 3:**
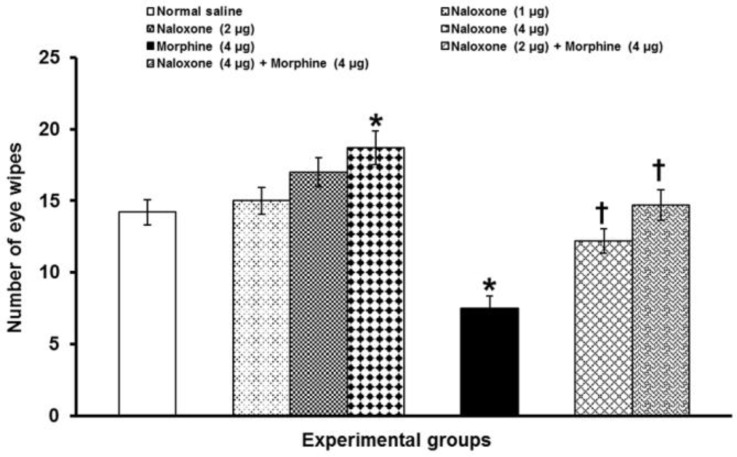
The effects of intra-PFN microinjection of naloxone alone and before morphine administration on corneal pain induced by topical corneal surface application of hypertonic saline. Data are the means ± SEM obtained from six rats. * *p* < 0.05 compared to normal saline microinjected group; and^ †^ *p *< 0.05 compared to morphine (4 μg) microinjected group

Alone bilateral intra-PFN microinjection of naloxone at doses of 1 and 2 μg per site did not alter corneal pain severity, whereas at a dose of 4 μg per site, it significantly (*p* < 0.05) increased the number of eye wipes. Prior microinjection of naloxone (2 and 4 μg per site) before morphine (4 μg per site) microinjection significantly (*p* < 0.05) prevented morphine-induced analgesia (Fig. 3). 

Bilateral microinjection of naloxonazine (0.5 μg per site) did not alter corneal pain severity, whereas at doses of 1 and 2 μg per site it significantly (*p* < 0.05) increased the number of eye wipes. 

**Fig. 4:**
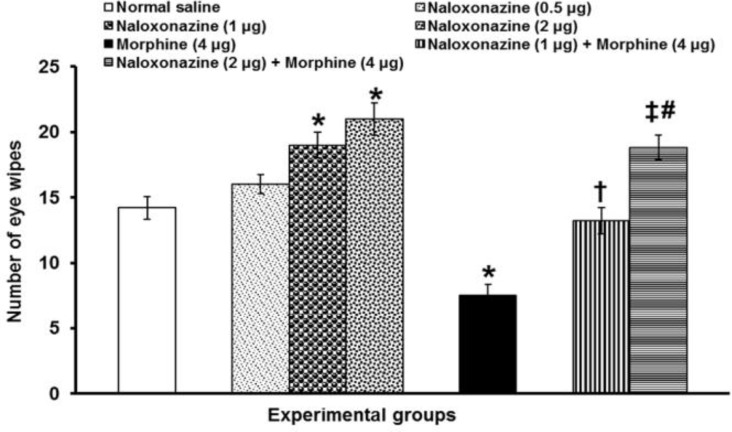
The effects of intra-PFN microinjection of naloxonazine alone and before morphine administration on corneal pain induced by topical corneal surface application of hypertonic saline. Data are the means ± SEM obtained from six rats. * *p* < 0.05 compared to normal saline microinjected group; ^†^ *p *< 0.05 compared to morphine (4 μg) microinjected group; ^‡^ *p *< 0.01 compared to morphine (4 μg) microinjected group; and ^#^ *p *< 0.05 compared to naloxonazine (4 μg) + morphine (4 μg) microinjected group

Prior microinjection of naloxonazine at doses of 1 μg per site (*p* < 0.05) and 2 μg per site (*p* < 0.01) significantly prevented the antin-ociceptive effect induced by 4 μg per site morphine. The inhibitory effect of naloxonazine (2 μg per site) was significantly (*p* < 0.05) more than that of 1 µg per site naloxonazine (Fig. 4). 

**Fig. 5:**
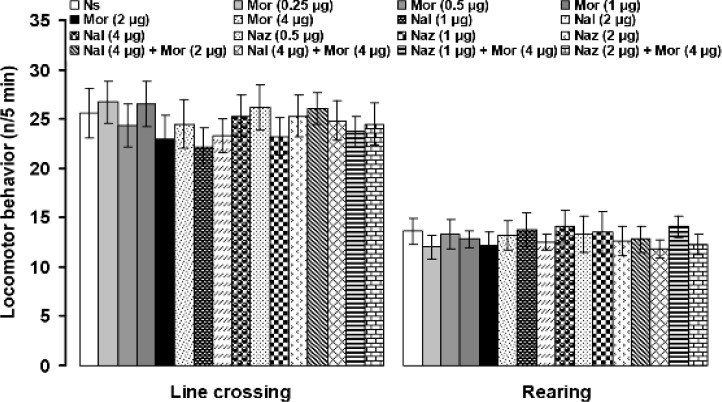
The effects of intra-PFN microinjection of normal saline (Ns), morphine (Mor), naloxone (Nal) and naloxonazine (Naz) in separate and combined treatments on the numbers of line crossing and rearing in an open-field test. Data are the means ± SEM obtained from six rats. There are no significant differences among treated groups

The numbers of line crossing and rearing were 25.6 ± 2.5 and 13.6 ± 1.3, respectively, after bilateral intra-PFN microinjection of normal saline. Microinjection of the all above-mentioned chemicals did not alter line crossing and rearing in the open-field test (Fig. 5). 

## Discussion

In the present study, local application of a 5 M NaCl solution in the cornea surface produced nociceptive behavior characterized by wiping of the eye with ipsilateral forepaw. Local corneal surface application of hypertonic saline has been frequently used to explore acute trigeminal pain mechanisms in rats.^6^^,^^12^^,^^19^^,^^20^ The wiping the eye with a forelimb, known as an eye-wiping test, has been used for the dry eye disease investigation, corneal hyperalgesia and exploring the peripheral and central mechanisms of trigeminal pain.^20^^-^^22^ Therefore, the results of the present study on eye wiping are in accordance with the above-mentioned investigations.

The present results showed an antinociceptive effect for morphine in the corneal pain at the PFN level of the thalamus. In addition, naloxone and particularly naloxonazine prevented this effect of morphine. These findings indicate that μ-opioid receptor may be involved in supraspinally-induced antinociception of morphine in acute trigeminal pain. Morphine, 7,8-didehydro-4,5-epoxy-17-methyl-(5α, 6α)-morphinan-3,6-diol, is the opium poppy principal alkaloid.^23^ Morphine, as a potent exo-genous opiate, is a gold standard analgesic commonly used to alleviate pain.^24^ Like the other exogenous opiate agents, morphine acts through μ-, δ- and κ-opioid receptors to produce antinociceptive effects at the peripheral, spinal and supraspinal levels of pain pathways.^25^^-^^27^ It binds to μ-opioid receptor with nearly two orders of magnitude greater affinity compared with δ- and κ-opioid receptors.^28^ Naloxone is a competitive antagonist of μ-, κ- and sigma-opioid receptors with higher affinity for the μ-opioid receptors.^24^ To clear the role of the PFN μ-opioid receptor involvement in morphine-induced antinociception, we used an specific antagonist of μ-opioid receptor in the present study. Autoradiographic and neurocehmial studies have identified that opioid receptors are widely distributed throughout brain with particularly high density in limbic structures, thalamic nuclei and cerebral cortex. In the thalamus, μ-opioid receptor is highly distributed in most thalamic nuclei, whereas κ-opioid receptor has a limited distribution and δ-opioid receptor is not found.^7^^,^^29^ Microinjection of μ-opioid receptor agonist, D-Ala^2^, N-Me-Phe^4^, glycinol^5^-enkephalin (DAMGO) into the centrolateral nucleus of thalamus, evoked a hyper-polarization response, which was blocked by application of a μ-opioid receptor antagonist, Cys^2^, Try^3^, Orn^5^, Pen^7^-amide.^30^ D-Pen2, D-pen5-enkephaline (a δ-opioid receptor agonist) and (±)-trans-U-50488 (a κ-opioid receptor agonist) had no effects.^30^ In previous study, a μ-opioid receptor antagonist, methylnaloxonium, into PFN, reversed the increased vocalization threshold induced by microinjection of morphine into the same site in the noxious tail shock model of pain in rats.^31^ In the formalin test of rats, microinjection of naloxone into the habenula, PFN and paraventricular nucleus of thalamus completely reversed the antinociceptive effect induced by microinjection of morphine into the same sites.^32^ In addition, microinjection of morphine into PFN increased the paw-lick latency in hot-plate test of nociception.^33^

Another nucleus of thalamus, submedius (sm) have also suggested to have important roles in morphine-induced antinociception. Microinjection of morphine into the sm nucleus of thalamus suppressed formalin-induced oro-facial pain in rats which was inhibited by prior microinjection of naloxone into the same site.^17^ In addition, anti-mechanical and anti-cold allodynia effects induced by microinjection of morphine into the sm were prevented by prior microinjection of naloxone into the same site.^34^ However, there is no report showing the effects of μ-opioid receptor specific antagonist microinjection into thalamic nuclei in modulation of acute trigeminal pain. The cornea is innervated by myelinated A-delta and unmyelinated C fibers that respond to chemical, thermal and mechanical stimuli of the cornea and send afferents via the ophthalmic branch of the trigeminal nerve to the trigeminal dorsal horn.^35^^,^^36^ Ascending corneal pain transmission is mediated primarily by pathways to either the thalamus or parabrachial nuclei.^37^^,^^38^ All the above-mentioned findings refer to this point that the μ-opioid receptor in thalamic nuclei may have important role in the supraspinal modulation of acute trigeminal pain.

In conclusion, the results of the present study showed an antinociceptive effect of morphine on corneal pain at the PFN level of the thalamus. Naloxone and naloxonazine inhibited the suppressive effect of morphine on corneal pain. Therefore, it can be assumed that μ-opioid receptor of the PFN may be involved in supraspinal pain modulation of morphine in corneal pain. 
